# Inter-Relations between Dietary Patterns and Glycemic Control-Related Biomarkers on Risk of Retinopathy in Type 2 Diabetes

**DOI:** 10.3390/nu16142274

**Published:** 2024-07-15

**Authors:** Yu-Ju Wu, Chih-Cheng Hsu, Shang-Jyh Hwang, Kun-Der Lin, Pi-Chen Lin, Ya-Fang Huang, Chien-Hung Lee, Chiao-I Chang, Meng-Chuan Huang

**Affiliations:** 1 Department of Nutrition and Dietetics, Kaohsiung Medical University Hospital, Kaohsiung Medical University, Kaohsiung 807378, Taiwan; erin@cc.kmu.edu.tw; 2 Graduate Institute of Medicine and Department of Public Health and Environmental Medicine, School of Medicine, College of Medicine, Kaohsiung Medical University, Kaohsiung 807378, Taiwan; s370002000@yahoo.com.tw; 3 Institute of Population Health Sciences, National Health Research Institutes, Miaoli County 350401, Taiwan; cch@nhri.org.tw (C.-C.H.); avon@nhri.edu.tw (Y.-F.H.); 4 Department of Health Services Administration, China Medical University, Taichung 4064040, Taiwan; 5 Department of Family Medicine, Min-Sheng General Hospital, Taoyuan 330056, Taiwan; 6 National Center for Geriatrics and Welfare Research, National Health Research Institutes, Yunlin 632007, Taiwan; 7 Division of Nephrology, Department of Internal Medicine, Kaohsiung Medical University Hospital, Kaohsiung Medical University, Kaohsiung 807378, Taiwan; kmuckd7901@gmail.com; 8 Division of Endocrinology and Metabolism, Department of Internal Medicine, Kaohsiung Medical University Hospital, Kaohsiung Medical University, Kaohsiung 807378, Taiwan; pichli@kmu.edu.tw; 9 The Lin’s Clinics, Kaohsiung 807057, Taiwan; berg.kmu@gmail.com; 10 Department of Public Health, College of Health Sciences, Kaohsiung Medical University, Kaohsiung 807378, Taiwan; cnhung@kmu.edu.tw

**Keywords:** diabetic retinopathy, glycated hemoglobin A1c, malondialdehyde

## Abstract

Diabetic retinopathy (DR), which can cause vision loss, may progress faster with poor glycemic control and oxidative stress. This study aims to examine how dietary patterns and glycemic control biomarkers relate to retinopathy risk in type 2 diabetes patients. In this study, we enrolled diabetic patients with retinopathy (DR) (*n* = 136) and without retinopathy (no DR) (*n* = 466) from a cohort of participants in the “Blood Pressure Control to Reduce the Risk of Type 2 Diabetic Nephropathy Study”. Hemoglobin A1c (HbA1c) and malondialdehyde were defined as elevated when their levels reached ≥8.5% and ≥2/3 (16.2 μm), respectively. Dietary data were collected by a food frequency questionnaire. Dietary patterns were identified by factor analysis. Elevated HbA1c was significantly correlated with increased risk of DR (OR: 2.12, 95% CI: 1.14–3.93, *p* = 0.017). In subjects with a high animal protein and processed food dietary pattern (≥highest tertile score) or a low vegetable intake pattern (<highest tertile score), elevated HbA1c was significantly associated with a 4.44-fold (95% CI: 1.34–14.68, *p* = 0.015), 3.96-fold (95% CI: 1.12–14.04, *p* = 0.033), and 2.57-fold (95% CI: 1.16–5.67, *p* = 0.020) increase in the risk of DR, respectively, compared to subjects with HbA1c levels < 8.5%. When stratifying subjects with a high animal protein pattern, higher MDA levels were significantly correlated with an increased risk of DR (OR: 2.93, 95% CI: 1.33–6.48, *p* = 0.008). Poor glycemic control increases the risk of retinopathy in patients with type 2 diabetes, and combined with diets low in vegetables and high in animal protein or processed food may exacerbate the risk of DR. The findings of this study should be further investigated in prospective studies.

## 1. Introduction

Diabetic retinopathy (DR) is a microvascular complication commonly affecting people with diabetes. If left untreated, it can lead to loss of vision or blindness [1,2]. Diabetic macular edema, which manifests as retinal thickening around the fovea, is primarily caused by persistent hyperglycemia and can occur at any stage during the development of DR [3]. Several risk factors contribute to the accelerated development of DR, including prolonged diabetes and poor diabetes control, marked by hyperglycemia and hypertension [2,3]. In patients with diabetes, because glucose combines with hemoglobin and other proteins, the measurement of hemoglobin A1c (HbA1c) levels is thought to be useful for assessing the control of blood sugar over the long term [4]. According to the Diabetes Control and Complications Trial (DCCT), rigorous treatment aimed at maintaining near-normoglycemic values significantly reduces the possibility of progression of retinopathy [5]. One meta-analysis has also reported that rigorous control of blood sugar can reduce its progression (RR = 0.77) and reduce the incidence of macular edema (RR = 0.66) in individuals with type 2 diabetes [6].

Oxidative stress can also contribute to the progression of DR since abnormal reactive oxygen species (ROS) production can injure tissues and nearby capillaries in the retina vessels [7]. Because polyunsaturated fatty acids are sensitive to oxidation, high polyunsaturated fatty acid (PUFA) concentrations in the retina contribute to the retina’s high vulnerability to oxidative stress [7]. Serum concentrations of malondialdehyde (MDA), a lipid peroxidation marker, have been correlated to higher levels of HbA1c in type 2 diabetes [8,9]. Blood biomarkers of lipid peroxidation, such as MDA [10] and urinary F2-isoprostanes [11], have also been positively correlated with DR or macular edema [12].

A systematic review of 29 case–control studies found higher MDA levels in those with DR [13]. Another study showed that poorly controlled diabetes patients had increased HbA1c and salivary MDA, indicating a correlation between MDA and glycemic control [9]. Regarding diet and DR, higher saturated fat intake increased the severity of DR in diabetic patients with HbA1C < 7.0% in a study conducted in Australia. Similarly, the JPHC-NEXT Eye Study found that total fat and saturated fat intake were associated with higher risks of DR. Both studies indicate that higher fat or saturated fat intake is linked to an increased risk and severity of diabetic retinopathy (DR) in diabetes. Dietary control during early diabetes may also help delay disease progression and the need for treatment. Although there have been some systematic reviews studying the effect of diet on progression to DR [14,15], very few studies simultaneously explored the relationship between glycemic control, oxidative stress indicators, and diets and the effect on the risk of retinopathy in a diabetic population. Therefore, in this cross-sectional study, we investigate the inter-relations between dietary patterns and metabolic biomarkers related to glycemic control and their association with retinopathy risk in patients with type 2 diabetes.

## 2. Materials and Methods

### 2.1. Study Design and Population

For this study, we enrolled participants from a cohort consisting of type 2 diabetes patients taking part in an intervention study (the BP4DM study, Clinicaltrials.gov NCT03477786) to investigate the effect of rigorous control of blood pressure on the development of nephropathy in this population. The study population of the BP4DM study originally consisted of 1250 type 2 diabetes patients aged 30–75 years with hypertension (>140/90 mmHg). The cohort was divided into two groups based on blood pressure control targets. The intervention group targeted blood pressure control at 120/75 mmHg, while the control group aimed for levels of 140/90 mmHg. Among these, 707 patients received multidisciplinary shared care diabetes program at the Departments of Endocrinology and Metabolism at Kaohsiung Medical University Hospital and Kaohsiung Municipal Ta-Tong Hospital (Kaohsiung, Taiwan) that were certified as Diabetes Health Promotion Institutions (DHPI) by Health Promotion Administration, Ministry of Health and Warfare, Taiwan. All of our patients were enrolled in a diabetes shared-care program established by the National Health Insurance in Taiwan (http://www.nhi.gov.tw/english; accessed on 10 October 2023.) and routinely scheduled to receive nutrition education for diabetes based on guidelines of the American Diabetes Association [16]. For current quality indicator of diabetic care of DHPI, HbA1c > 8.5% is listed as status of glycemic control indicator [17]. Of 707 subjects, 602 patients had complete data of age, gender, disease duration, education, smoking/drinking/exercise status, intervention or control group in original cohort study, BMI, MDA, HbA1c, and food frequency questionnaire were included in this analysis. In that study, patients were excluded if they had type 1 diabetes or gestational diabetes or had an HbA1c of >10%, albuminuria > 300 mg/g, non-diabetes-associated albuminuria, or an estimated glomerular filtration rate (eGFR) of <30 mL/min/1.73 m^2^. They were also excluded if they had kidney stones >0.5 cm or a history of myocardial infarction or other cerebrovascular events, cancer treated within 3 years, or liver cirrhosis, or if they had received foot amputation or dialysis.

In the current study, post hoc analysis was performed on second-year cross-sectional data collected between March 2014 and January 2018. Subjects with DR were diagnosed following criteria established by the American Academy of Ophthalmology. The degree of DR was classified into five stages based on the criteria of the Early Treatment Diabetic Retinopathy Study (ETDRS) [18]. The severity of DR was categorized as no DR, mild nonproliferative DR (NPDR), moderate NPDR, severe NPDR, and proliferative DR (PDR). In our study, the DR group (n = 136) consisted of mild, moderate, and severe NPDR, as well as PDR, while no NPDR was considered the no-DR group (n = 466). Mydriasis-free machines (Canon CR-2 digital fundus camera, Tokyo, Japan and Kowa nonmyd7, Tokyo, Japan) were used to perform fundus color photography examination. The diagnosis of DR was confirmed by endocrinologists or ophthalmologists. The protocol for this study was approved by ethics review committees of Taiwan’s National Health Research Institutes (EC1020201, approval date: 1 April 2013) and Kaohsiung Medical University Hospital (KMUHIRB-E(II)-20180233, approval date: 20 July 2018). All patients signed written informed consent forms.

### 2.2. Demographic and Clinical Data Collection

The height, weight, waist circumference, and blood pressure of each patient were assessed by trained research assistants. A questionnaire was used to collect almost all patient data, including age, sex, education, duration of diabetes, total household income, diabetes care status, lifestyle factors (smoking, alcohol drinking, betel nut chewing, exercise habits, and dietary habits), past medical history, drug use history, and physical and mental health status. Fasting blood was drawn from a superficial forearm vein for laboratory analysis of fasting blood glucose, cholesterol, triglyceride, high-density lipoprotein cholesterol (HDL-C), low-density lipoprotein cholesterol (LDL-C), HbA1c, blood urea nitrogen (BUN), serum creatinine and uric acid concentrations. All measurements were assessed in laboratories belonging to the Department of Medical Technology at Kaohsiung Medical University Chung-Ho Memorial Hospital and Kaohsiung Municipal Ta-Tung Hospital, which is operated by Kaohsiung Medical University Chung-Ho Memorial Hospital.

### 2.3. Plasma Malondialdehyde (MDA) and 8-Isoprostanes

Fasting blood was sampled at recruitment and stored under −80 °C for additional biomarker analyses. Our analysis of MDA concentrations was assessed by colorimetric thiobarbituric acid reactive substance (TBARS) assay (Cayman Chemical Company, Ann Arbor, MI, USA). The TBARS assay is a well-established method for the screening and monitoring of lipid peroxidation. Formation of MDA-TBA adduct was produced under acidic conditions at high temperatures (90–100 °C) and was measured calorimetrically at 530 nm [19]. The application of the 66th percentile was considered the cutoff for high MDA for this study. To measure the 8-isoprostanes concentration, we used an 8-isoprostane ELISA Kit (Cayman Chemical Company, Ann Arbor, MI, USA), a method based on the competition between 8-isoprostane and an 8-isoprostane-acetylcholinesterase conjugate for a small number of sites for 8-isoprostane-specific rabbit antiserum-binding [20].

### 2.4. Dietary Assessment

A validated semi-quantitative food frequency questionnaire (FFQ) with 45 questions structured to evaluate the frequency of food group intake, serving sizes, and some eating habits was used [21]. The participants answered questions regarding the frequency at which they consumed food items belonging to certain food groups during the six months leading up to the interview. That questionnaire offered nine frequency options, ranging from “almost never” to “four to six times per day”. The intake of certain food groups (Chinese staple foods, bread and cereals, light- or dark-colored vegetables, root vegetables, fresh fruits, canned juices, red or white meats, marine or pound fish, eggs, soy products, milk, and other dairy products, etc.), were expressed in common portion sizes consumed per day, week, or month. We converted each participant’s daily portions into weekly equivalents and estimated his or her daily energy intake. Energy was estimated when calculating the daily intake of the different food groups as well as cooking oil and these daily intakes were converted into daily intakes of calories and macronutrients per person [21].

### 2.5. Statistical Analysis

Descriptive data are presented as n (%) or mean ± standard deviation (SD). DR and no DR group demographic and clinical characteristics were analyzed using Pearson chi-squared tests and Student’s *t*-tests. Dietary patterns were identified by factor analysis of each patient’s responses to the FFQ. Uncorrelated factors, here referred to as dietary patterns, were analyzed using principal component extraction and varimax rotation. Food items were considered important contributors to the pattern if they had a factor loading ≥ ±0.3, calculated by multiplying the food items by their corresponding factor loadings and summation. The independent association between HbA1C, MDA and risks of DR was tested by multiple logistic regression adjusting for age (<65, ≥65 years), gender, diabetes duration (<15, 15–30, ≥30 years), energy intake, eGFR (<60, ≥60 mL/min/1.73 m^2^), smoking status (yes, no), drinking status (yes, no), exercise status (yes, no), education (<6, ≥6 years), intervention or control group in the original cohort study (targeting blood pressure control at 120/75 or 140/90 mmHg). All statistical operations were performed using SPSS (version 22.0; SPSS, Armonk, NY, USA). Significance was set at *p* < 0.05.

## 3. Results

As shown in Table 1, a summary of patient characteristics revealed that those with DR had a significantly longer average duration of diabetes (16.6 ± 8.7 years) compared to those without DR (11.2 ± 7.7 years; *p* < 0.001). Compared to patients who did not have DR, those who had it had significantly higher systolic blood pressure (143 ± 18 mmHg vs. 139 ± 17 mmHg; *p* = 0.003), HbA1c levels (7.6 ± 1.1% vs. 6.9 ± 0.9%; *p* < 0.001), and MDA levels (15.9 ± 5.8 µm vs. 14.5 ± 4.8 µm; *p* = 0.006). Both groups were similar with regard to sex, age, education level, smoking, drinking, exercise, BMI, serum triglyceride, uric acid, BUN, and creatinine levels.

Using multiple logistic regression analysis adjusted for age, gender, diabetes duration, energy intake, eGFR, smoking, drinking, exercise, education, and intervention or control group in the original cohort study, we found an increased risk of DR in those with higher levels of HbA1c (>8.5% vs. <8.5%, OR: 2.12, 95% CI: 1.14–3.93, *p* = 0.017). Levels of MDA (≥16.2 μM vs. <16.2 uM, OR: 1.38, 95% CI: 0.89–2.12, *p* = 0.147) did not relate to risks of DR. Additionally, those with a diabetes duration lasting 15–30 years (OR: 3.22; 95% CI: 2.07–5.04, *p* < 0.001) and >30 years (OR: 6.59; 95% CI: 2.85–15.22, *p* < 0.001) had increasing risks of DR, compared to those who had diabetes less than 15 years (Table 2).

The results of our factor analysis identified three dietary patterns (Table 3). The first was a high animal protein dietary pattern characterized by the frequent consumption of red or white meat, marine or freshwater fish, fatty meats, smoked and processed meats, and seafood. The second was a highly processed diet pattern, characterized by the frequent consumption of gluten products, dips, thickeners, processed soy, fried foods, canned foods, fermented products, low-nitrogen starches, outside meals (snacks), and soy products. The third was a high vegetable dietary pattern characterized by the frequent consumption of dark or light-colored vegetables.

Table 4 shows the associations of dietary pattern, HbA1c level, and MDA level with the risk of retinopathy in patients with type 2 diabetes after adjustment. The cutoff point separating high from low intake of certain food patterns was set at the highest tertile dietary factor scores (≥2/3) (high intake) and <2/3 (low intake), respectively. In those with low intakes of animal protein/processed food and high intake of vegetable dietary patterns, HbA1c level and MDA level did not appear to correlate with risks of DR. Conversely, in those with high intakes of animal protein and processed food patterns, high HbA1c (≥8.5)-associated risk of DR increased to 4.44 (95% CI: 1.34–14.68, *p* = 0.015) and 3.96 (1.12–14.04, *p* = 0.033). While an HbA1c of ≥8.5 was significantly positively correlated with the risk of DR in subjects with low vegetable intake pattern (OR: 2.57, 95% CI: 1.16–5.67, *p* = 0.020), compared to HbA1c < 8.5, this association was not observed in those with high vegetable diet factor scores. Regarding dietary animal protein, MDA levels ≥16.2 μM were significantly associated with increased risk of DR (OR: 2.93, 95% CI: 1.33–6.48, *p* = 0.008) when compared with MDA levels <16.2 μM. This association was only observed in those with high dietary animal protein scores. Pearson correlation analysis was used to test the correlations between dietary factor scores and MDA; no correlations were found between the two. This study did not find that either high or low dietary scores for processed and vegetable food patterns increased DR risks associated with MDA.

## 4. Discussion

This study found an association between higher HbA1c (≥8.5%) levels and the risk of retinopathy in patients with type 2 diabetes. By stratifying diabetic subjects with low intakes of animal protein/processed food and high intake of vegetable dietary patterns, HbA1c level, and MDA level did not appear to correlate with risks of DR. It appeared that our diabetes patients with poor glycemic control (HbA1c ≥ 8.5%) combined with unhealthy dietary patterns as high animal protein and processed food pattern intakes showed increased risks of DR compared to subjects with HbA1c < 8.5%. Our results revealed that poorer glycemic status in combination with unhealthy dietary patterns may augment the risk of retinopathy in our patient population.

Retinopathy is a specific and early clinical complication associated with diabetes and one of several diagnostic indicators of diabetes mellitus [22]. Multiethnic datasets derived from the DETECT-2 international pooling collaboration project [23] have also demonstrated the usefulness of HbA1c levels in relation to retinopathy to diagnose possible diabetes. Moreover, one recent meta-analysis [24] has found an association between poor glycemic control (higher HbA1c levels) and the risk of DR in diabetic patients, compared to those with good glycemic control (lower HbA1c levels) (OR: 1.25, 95% CI: 1.14–1.38). Few studies have reported on the possible interplay among HbA1c, oxidative stress, and retinopathy in diabetes. One prospective 3-year hospital-based study in India [25] recruiting 22 type 2 diabetes patients without retinopathy, 21 with non-proliferative DR, 22 with proliferative DR, and 22 age/sex-matched controls found a progressive increase in MDA, a lipid peroxidation product, starting in diabetes patients without retinopathy to those who had developed it. Furthermore, one study of cases with type 2 diabetes (n = 44) and healthy controls (n = 44) [9] conducted in Iran found that the cases had higher MDA (saliva) and HbA1c than the controls. A systematic review and meta-analysis [13] of 29 case–control studies totaling 1680 people with DR and 1799 without, reported significantly higher concentrations of circulating MDA in those with DR than in those without (*p* < 0.001). Another study found patients with poor (8.0 ≥ HbA1C% ≤ 10.0) and very poor (HbA1C > 10.0%) controlled diabetes trends to have concomitant increases in HbA1c (*p* = 0.001) and salivary MDA (*p* = 0.027), indicating a correlation between MDA and glycemic control [9]. When oxidative stress is not controlled, there is an increase in MDA in people with DR. A review [26] has reported that when oxidative stress goes uncontrolled in people with DR, lipids, particularly PUFAs are oxidized by the hydroxyl radicals, producing a variety of bioactive aldehydes. Another study has reported that secondary oxidized products, including MDA, can form adducts with cellular proteins, and has suggested that protein carbonylation may activate other pathways or factors not only contributing to the progression of diabetes but also injuring retina structure and function [27]. Based on our study results and previous investigations [9,24,25], poor glycemic control may exacerbate lipid peroxidation and accelerate the progression to retinopathy in patients with type 2 diabetes.

Poor glycemic control and poor blood pressure control are two key risk factors for microvascular complications associated with retinopathy in patients with type 2 diabetes, so lifestyle interventions, including diet and exercise modification, are beneficial for reducing the risk of retinopathy [28,29]. A 2018 systematic review of 31 studies (3 interventional and 28 observational) studying the relationship between dietary intake and DR [30] found that the Mediterranean diet may protect against incident DR, emphasizing the importance of a Mediterranean diet characterized as a diet high in dietary fiber and antioxidants, low in calories, and with a protein source of mostly oily fish. The effect of diet is likely driven by the increased consumption of vegetables, fruits, and nuts [30]. A recent review also concluded that both the Mediterranean diet and plant-based diets, replete with vegetables, whole grains, legumes, fruits, and nuts and low in animal products and processed foods, can protect against DR and vision loss from DR [31]. Similar to studies reporting an association between unhealthy foods or nutrients and retinopathy [32], our study found that unhealthy dietary patterns (more animal proteins, more processed foods, and fewer vegetables) combined with poor glycemic control correlated significantly with increased risks of retinopathy in diabetic patients. However, the association between high animal-based food intake and DR could be based on reverse causality. Patients with higher disease load tend to a more low-carb diet, thus, eating more animal-based foods.

It has been suggested that dyslipidemia and corresponding fatty acids may affect the development of DR in diabetes [33]. A study [34] conducted within the Japan Public Health Center-based Prospective Study for the Next Generation (JPHC-NEXT) Eye Study (n = 647 diabetes with 100 DR) found that total fat and saturates correlated significantly with the likelihood of DR presence, even after adjusting for potential factors (OR: 2.61, *p* trend = 0.025 and OR: 2.40, *p* trend = 0.013). In Australia, a cross-sectional study with 379 diabetic patients also showed that higher saturated intake was associated with an increased likelihood and severity of DR in patients with HbA1C < 7.0%, but not those with poorly controlled diabetes (HbA1C > 7.0%) [35]. Our results were similar to the results of the Australian study; harmful effects of hyperglycemia could interact with foods high in saturated fat, such as animal protein, and may subsequently affect the risks of DR. In addition to the impact of saturated fat, intake of meat and meat products can increase gastrointestinal ROS [36]. Many products, including cytotoxic reactive aldehydes, ketones, and epoxides, are generated from the hydroperoxide decomposition of lipids [37]. We were not able to separate the effects of animal proteins from different food sources based on our food patterns. However, other investigations among Caucasians, Asians in Singapore, and Indians have consistently reported that fish consumption reduces the risk of retinopathy in patients with type 2 diabetes [38,39,40]. Diets deficient in *n*-3 PUFA have been found to alter the structure and function of the retina in patients with age-related macular degeneration, possibly because of decreased amounts of *n*-3 PUFA, which have been found to have anti-inflammatory properties and inhibit neovascularization and decrease retinal pigment epithelial oxidative damage [41,42].

In addition to poor glycemic control, this study found elevated MDA (OR: 2.93; 95% CI: 1.33–6.48, *p* = 0.008) combined with an unhealthy diet including a high intake of animal protein to be associated with a higher risk of DR. It has been suggested that meat and meat products undergo oxidative changes during storage, processing, digestion, and metabolism, making them potential sources of oxidizing agents. These changes take place from the moment the animal is slaughtered, when the conversion of the muscle into meat begins to form oxidizing compounds [43]. Additionally, a high intake of meat and meat products can promote the formation of ROS in the gastrointestinal tract [36], and many of these products are generated from lipid hydroperoxide decomposition, such as reactive aldehydes, ketones, and epoxides, which are cytotoxic [37]. Further, during the Tehran Lipid and Glucose Study (TLGS), a prospective survey was administered to 400 residents with metabolic syndrome in Tehran [44]. That study, analyzing the regression coefficient for MDA within different quintiles of dietary patterns and adjusting for potential confounders, found a significant positive association between serum MDA levels and unhealthy dietary patterns (β = 0.387, *p* = 0.0001). It suggested that regularly consuming a diet rich in fruits and vegetables could bring about a reduction in oxidative stress, whereas regular consumption of an unhealthy pattern, i.e., a diet high in fast and processed foods, could worsen oxidative stress in residents with metabolic syndrome [44].

This study had some limitations. One limitation is that it was a cross-sectional study, a design that cannot be used to establish causality. Another limitation is its small sample size. Another limitation is that we only measured a small number of oxidative biomarkers at a single time point, and multiple measurements would have provided a more precise estimate. Still, another limitation is that there may be other unmeasured confounding effects that we did not adjust for in our multivariable regression analyses. Although this study is cross-sectional, its results demonstrate some strengths. Given the limited research on the interplay among glycemic control, oxidative stress markers, dietary habits, and their combined effects on retinopathy risk in diabetic populations, the findings of this study hold particular importance. Specifically, this study utilized a validated FFQ [21] to define food patterns and assess their roles in the risk of DR. While several systematic reviews have focused on examining the impact of individual food items or nutrients on the progression of DR [14,15], assessing overall consumption patterns may better reflect the diet’s impact due to the interrelations or combined effects of these dietary components.

## 5. Conclusions

This study concluded type 2 patients with poor glycemic control combined with dietary patterns of low vegetable, high animal protein, and processed food may have an increased risk of retinopathy. Additionally, elevated MDA levels combined with a high animal protein diet also pose a higher risk of DR. Therefore, promoting a healthier diet, such as eating more vegetables and consuming less meat and processed foods, is appropriate for reducing HbA1c and possibly improving oxidative status in people with type 2 diabetes. Prospective studies are needed to establish causal relationships and further elucidate the role of prudent diet in modulating the risk of retinopathy.

## Figures and Tables

**Table 1:** Demographic and clinical characteristics of type 2 diabetes with retinopathy (DR) or without retinopathy (no DR) ^1^.

	No DR (n = 466)	DR (n = 136)	*p* ^2^
Age	66.0 ± 8.5	65.2 ± 8.5	0.350
Diabetes duration (year)	11.2 ± 7.7	16.6 ± 8.7	<0.001
Male (%)	234 (50.2%)	74 (54.4%)	0.389
Education ≤ 6 y (%)	141 (30.3%)	45 (33.1%)	0.530
Current smoker (%)	74 (15.9%)	19 (14.0%)	0.588
Alcohol drinker (%)	45 (9.7%)	13(9.6%)	0.973
Exercise habits (%)	324 (69.5%)	100 (73.5%)	0.368
Systolic BP (mmHg)	139 ± 17	143 ± 18	0.003
Diastolic BP (mmHg)	78 ± 11	79 ± 11	0.698
BMI (kg/m^2^)	26.7 ± 4.0	26.5 ± 4.1	0.536
Hemoglobin A1c (%)	6.9 ± 0.9	7.6 ± 1.1	<0.001
Triglycerides (mg/dL) ^3^	134.7 ± 73.9	136.4 ± 122.1	0.847
Uric acid (mg/dL) ^3^	5.7 ± 1.4	5.9 ± 1.6	0.189
BUN (mg/dL) ^3^	15.5 ± 5.3	16.0 ± 6.4	0.438
Creatinine (mg/dL)	0.9 ± 0.3	0.9 ± 0.3	0.545
eGFR (mL/min/1.73 m^2^)	88.3 ± 27.2	87.2 ± 29.5	0.702
<60 ≥60	63 (73.3%) 403 (78.1%)	23 (26.7%) 113 (21.9%)	0.320
Malondialdehyde (µM)	14.5 ± 4.8	15.9 ± 5.8	0.006
8-Isoprostane (pg/mL) ^3^	188.4 (81.8–343.9)	190.6 (74.5–302.4)	0.570

DR, diabetic retinopathy; eGFR, glomerular filtration rates; BMI, body mass index. ^1^ Values are presented as mean ± SD, and 8-isoprostane is shown as median (25th and 75th percentiles). ^2^ *t*-test or chi-square test was used to test differences between DR and no DR. ^3^ There were 4, 38, 155, and 22 missing values for triglyceride, uric acid, BUN, and 8-isoprostane, respectively.

**Table 2:** Results of odds ratios (OR) for risks of diabetic retinopathy (DR) using multivariable logistic regression analysis.

Risk Factor	No DR N (%)	DR N (%)	Crude Model OR	95% CI	*p*	Adjusted Model OR ^1^	95% CI	*p*
Sex								
Male	234 (50.2)	74 (54.4)	1			1		
Female	232 (49.8)	62 (45.6)	0.85	0.58–1.24	0.389	0.84	0.53–1.34	0.476
Age (year)								
<65	185 (39.7)	58 (42.6)	1			1		
≥65	281 (60.3)	78 (57.4)	0.89	0.60–1.30	0.538	0.60	0.38–0.95	0.028
Duration of diabetes (year)								
<15	347 (74.5)	63 (46.3)	1			1		
15–30	105 (22.5)	58 (42.5)	3.04	2.00–4.62	<0.001	3.22	2.07–5.04	<0.001
≥30	14 (3.0)	15 (11.0)	5.90	2.72–12.83	<0.001	6.59	2.85–15.22	<0.001
Smoking status								
No	392 (84.1)	117 (86.0)	1			1		
Yes	74 (15.9)	19 (14.1)	0.86	0.50–1.48	0.588	0.85	0.46–1.58	0.612
Drinking status								
No	421 (90.3)	123 (90.4)	1			1		
Yes	45 (9.7)	13 (9.6)	0.97	0.52–1.89	0.973	0.96	0.46–2.02	0.961
Exercise status								
No	142 (30.5)	36 (26.5)	1			1		
Yes	324 (69.5)	100 (73.5)	1.22	0.79–1.87	0.369	1.24	0.78–1.98	0.361
Malondialdehyde (uM)								
<16.2	320 (68.7)	77 (56.6)	1			1		
≥16.2	146 (31.3)	59 (43.4)	1.68	1.14–2.48	0.009	1.38	0.89–2.12	0.147
Hemoglobin A1c (%)								
<8.5	443 (79.2)	116 (81.7)	1			1		
≥8.5	34 (7.1)	26 (18.3)	2.72	1.55–4.78	<0.001	2.12	1.14–3.93	0.017

DR, diabetic retinopathy; OR, odds ratio; CI, confidence interval. ^1^ Adjusted for age (<65, ≥65 years), sex, duration of diabetes (<15, 15–30, ≥30 years), energy intake, eGFR (<60, ≥60 mL/min/1.73 m^2^), smoking (yes, no), drinking (yes/no), exercise (yes/no), education (<6, ≥6 years), cohort study group (targeting blood pressure control at 120/75 or 140/90 mmHg) by multiple logistic regressions.

**Table 3:** Factor loading matrix for the three major dietary patterns identified from the food frequency questionnaire ^1^.

	Factor 1 Animal Protein	Factor 2 Processed Food	Factor 3 Vegetables
White meat	0.654	0.034	−0.143
Red meat	0.622	0.061	−0.195
Marine fish	0.599	−0.116	0.192
Freshwater fish	0.577	−0.191	0.210
Fatty meats and skin	0.566	−0.073	0.092
Smoked meat, salted meat	0.428	0.177	−0.176
Seafood	0.418	0.276	−0.219
Fresh fruits	0.177	0.126	0.055
Low calorie snacks	0.087	0.021	−0.044
Gluten products	−0.090	0.486	−0.005
Sause use	0.061	0.460	−0.039
Starch/thickened soup and food	0.037	0.445	−0.016
Processed soy products	−0.224	0.397	0.062
Fried food	0.063	0.388	−0.242
Canned meats	0.056	0.386	−0.168
Pickled vegetables	0.127	0.364	−0.009
Fermented products	0.029	0.350	0.062
Low nitrogen staple foods	0.145	0.335	−0.117
Eating out	0.056	0.334	−0.197
Soy products	0.017	0.313	−0.102
Seeds and nut	0.154	0.291	0.006
Chinese pastries and foreign pastries	−0.003	0.272	0.060
Chinese staple food	−0.047	0.263	0.176
Sugar substitute	−0.060	0.233	0.076
Eggs	0.140	0.220	−0.173
Root food	0.060	0.201	0.008
Sugar-free tea	0.065	0.179	−0.022
Handshake beverages	−0.071	0.174	−0.104
Juice	−0.063	0.172	−0.028
Milk, yogurt	0.070	0.106	0.062
Light-colored vegetables	0.176	0.093	0.872
Dark-colored vegetables	0.157	0.126	0.867
Bread	0.102	−0.002	−0.204
Processed dairy products	0.077	0.080	−0.204
% Variance explained	7.3%	7.0%	6.1%

^1^ Omitted from the table were food items or groups with factor loadings < ±0.3 for all dietary patterns.

**Table 4:** Independent associations between hemoglobin A1C and malondialdehyde and risks of diabetic retinopathy (DR) in strata of low or high factors of three dietary patterns by multiple logistic regression analysis.

		Crude Model	*p*	Adjusted Model ^1^	*p*
		OR (95% CI)		OR (95% CI)	
Model 1: <2/3 animal protein diet factor score (n = 415)					
Malondialdehyde (μM)	≥16.2 vs.<16.2	1.20 (0.73–1.94)	0.482	1.10 (0.87–1.91)	0.734
Hemoglobin A1c (%)	≥8.5 vs.<8.5	2.13 (1.08–4.21)	0.029	1.91 (0.87–4.19)	0.107
Model 2: ≥2/3 animal protein diet factor score (n = 197)					
Malondialdehyde (μM)	≥16.2 vs.<16.2	3.27 (1.65–6.46)	0.001	2.93 (1.33–6.48)	0.008
Hemoglobin A1c (%)	≥8.5 vs.<8.5	5.00 (1.75–14.32)	0.003	4.44 (1.34–14.68)	0.015
Model 3: <2/3 processed food factor score (n = 401)					
Malondialdehyde (μM)	≥16.2 vs.<16.2	1.69 (1.07–2.72)	0.026	1.50 (0.89–2.52)	0.131
Hemoglobin A1c (%)	≥8.5 vs.<8.5	2.48 (1.27–4.84)	0.008	1.78 (0.84–3.78)	0.136
Model 4: ≥2/3 processed food factor score (n = 201)					
Malondialdehyde (μM)	≥16.2 vs.<16.2	1.60 (0.78–3.30)	0.199	1.43 (0.59–3.46)	0.434
Hemoglobin A1c (%)	≥8.5 vs.<8.5	3.33 (1.18–9.39)	0.023	3.96 (1.12–14.04)	0.033
Model 5: <2/3 vegetables diet factor score (n = 402)					
Malondialdehyde (μM)	≥16.2 vs.<16.2	1.65 (1.01–2.68)	0.044	1.25 (0.73–2.16)	0.475
Hemoglobin A1c (%)	≥8.5 vs.<8.5	3.10 (1.54–6.24)	0.002	2.57 (1.16–5.67)	0.020
Model 6: ≥2/3 vegetables diet factor score (n = 200)					
Malondialdehyde (μM)	≥16.2 vs.<16.2	1.73 (0.89–3.34)	0.105	1.61 (0.75–3.46)	0.222
Hemoglobin A1c (%)	≥8.5 vs.<8.5	2.14 (0.83–5.52)	0.116	2.00 (0.66–6.00)	0.218

DR, diabetic retinopathy; OR, odd ratio; CI, confidence interval. ^1^ Adjusted for age (<65, ≥65 years), sex, duration of diabetes (<15, 15–30, ≥30 years), energy intake, eGFR (<60, ≥60 mL/min/1.73 m^2^), smoking (yes, no), drinking (yes/no), exercise (yes/no), education (<6, ≥6 years), cohort study group (targeting blood pressure control at 120/75 or 140/90 mmHg) by multiple logistic regressions.

## Data Availability

The data presented in this study are available on request from the corresponding author. The reason for the restriction is that the data are not publicly available due to privacy and ethical restrictions.
